# Temporal trend of research related to gun violence from 1981 to 2018 in the United States: a bibliometric analysis

**DOI:** 10.1186/s40621-020-0235-6

**Published:** 2020-03-23

**Authors:** Lung-Chang Chien, Maxim Gakh, Courtney Coughenour, Ro-Ting Lin

**Affiliations:** 1 Department of Environmental and Occupational Health, School of Public Health, University of Nevada, Las Vegas, 4700 S. Maryland Pkwy, Suite 335, Las Vegas, Nevada 89119 USA; 2 Department of Occupational Safety and Health, College of Public Health, China Medical University, No. 91, Xueshi Road, North District, Taichung City, 404 Taiwan

**Keywords:** Gun violence, Injury, Bibliometric analysis, Trend

## Abstract

**Background:**

We aimed to evaluate the variation in gun violence-related research in the US over time to determine if there are meaningful changes in frequency of research at certain time points. Related publications were searched from the Web of Science.

**Methods:**

We searched articles from Web of Science to collect publication data of gun violence research in three disciplines (clinical sciences, life sciences, and social behavior sciences) from 1981 to 2018. The joinpoint regression approach was applied to evaluate the trend of publication ratio. We also adopted the generalized additive mixed model to compare the publication ratio among the three research disciplines.

**Results:**

During the study period, each research discipline had a significant decrease in publication ratios, especially social behavioral sciences from 2001 to 2011, with an annual percentage change = − 9.77% (95% CI = − 13.45, − 5.93; *p*-value < .0001). After combining the three research disciplines, the average change of the publication ratio was significantly increased 9.18% (95% CI = 6.42, 12.01; p-value < .0001) per year from 1981 to 2018. Compared to social behavioral sciences, both clinical sciences and life sciences had a significantly smaller publication ratio.

**Conclusions:**

Gun violence research exhibited a significant downward trend in publications in the early 2000s, which may be attributed at least in part to limited federal funding, but the publication ratio increased since the 2010s. To enhance the amount of peer-reviewed gun violence research so that research-informed gun violence interventions are more likely to succeed, decision-makers should keep supporting quality research.

## Background

Gun violence is a critical public health issue in the US and contributes significantly to premature morbidity and mortality. In 2017 there were nearly 40,000 firearm related deaths (Pilkington 2018). Firearm death rates in the US far surpass those of all other populous high income OECD countries; US overall firearm death rates are 10 times higher, firearm suicide rates are 8 times higher, and firearm homicide rates are 25 times higher (Grinshteyn and Hemenway 2016). Furthermore, gun violence has substantial economic consequences, with medical and work-loss costs alone for fatal and non-fatal firearm injuries estimated at over $45 billion annually (Centers for Disease Control and Prevention 2010), and these costs do not even take into account lost quality of life and any physical, social, or emotional toll on families and loved ones.

Firearm ownership has been associated with increased rates of intentional and unintentional firearm deaths. State-level gun ownership rates have been found to be a strong correlate of firearm homicide rates when using various national datasets (Siegel et al. 2013; Siegel et al. 2014). Additionally, research posits that states that have more permissive concealed-carry gun laws have higher homicide rates (Siegel et al. 2017). Firearm ownership, access and storage practices are a risk factor for suicide (Brent et al. 1993; Brent et al. 1991; Cummings et al. 1997; Bukstein et al. 1993; Kellermann et al. 1992; Grossman et al. 2005; Miller et al. 2002). A meta-analysis of 16 observational studies concluded that access to firearms is associated with risk for completed suicide and being the victim of homicide (Anglemyer et al. 2014). Various studies have also linked firearm access to unintentional firearm deaths, particularly among youth (Miller et al. 2002; Grossman et al. 1999; Levine and McKnight 2017).

Despite what we know about gun violence, many related topics remain only partially understood and therefore not fully actionable. For example, a Community Guide systematic review focused specifically on law-based interventions to gun violence concluded in 2005 that there was insufficient evidence to determine whether firearm laws were effective interventions (Hahn et al. 2005). More recently, an expert-crafted research agenda around gun violence calls for a need to better understand, among other issues, gun violence characteristics, ways to prevent gun-related injuries, and risks and protective factors of gun violence (Council NR 2013).

The persistence of such important questions in this area may not be surprising in light of patterns of gun violence research. It may be explained, at least in part, by a chilling effect created by Congressional efforts that began in 1996 to limit funding for gun-violence research at and supported by federal agencies, and in particular the Centers for Disease Control and Prevention (CDC) that especially affected health-related disciplines (Stark and Shah 2017; Rubin 2016; Kellermann and Rivara 2013; Ladapo et al. 2013). In the context of health-related research, studies have shown under-funding of gun violence research relative to its health consequences. For example, Stark and Shah demonstrated that the expected rates of publication and federal funding for gun violence research appears low compared to other causes of death (2017). As Kellermann and Rivara articulate: “The nation might be in a better position to act if medical and public health researchers had continued to study these issues” (2013).

Bibliometric analysis, frequently used to assess journal article output, is a sub-field of infometrics, used to measure publication outputs and includes assessing the amount of research in a particular field (Ismail et al. 2012). Bibliometric research output information can be a proxy for how much research is being conducted on a particular topic and can also be used to help propel progress in a research field (Ismail et al. 2012). In the area of gun violence research, existing bibliometric research indicates that gun violence research article publication has varied by year since the 1970s, with an increase between 1985 and 1999 and then a level period through 2012 (Alcorn 2017). Relative to the overall growth of scientific literature, however, there appears to be a decline in gun violence publications between 1998 and 2012 (Alcorn 2017).

While these general patterns emerge, what is less clear is whether there are points in time in which there are meaningful changes in frequency of publication. These points are important to identify because they can help pinpoint what periods of time should be probed further to determine why the incidence of publication changed. Determining changes in publication incidence can help understand research around gun violence, the findings of which can help identify evidence-informed interventions to reduce gun violence and thereby suicide and homicide. As a result, we aimed to evaluate the variation in gun violence-related publication in the US over time to identify whether there are meaningful changes in frequency of publications at certain time points and to consider whether covariates of gun-violence are associated with changes in publication ratios.

## Methods

### Data source

We retrieved all publication information from the Web of Science Core Collection database (Clarivate Analytics). We found these publications through a series of key terms searches. First, we set up 10 primary keywords to capture gun concepts (i.e., gun, handgun, firearm, rifle, shotgun, pistol, revolver, semi-automatic, bullet, and ammunition). Then, we selected five secondary keyword sets to ensure that the articles collected through primary keyword searches actually focused on researching elements of gun violence that related to its health risks, health consequences, and policy interventions: violence (10 keywords), perpetrator (6 keywords), health outcome (10 keywords), risk factor (11 keywords), and law (39 keywords) concepts. Researchers generated these keywords with the help of online databases, such as Medical Subject Headings, which is a vocabulary thesaurus used for indexing articles in PubMed, and PubReMiner, which links to the PubMed literature database for text mining. Detailed keywords for each category are provided Table 1. Finally, a total of 770 (10 × (10 + 6 + 10 + 11 + 39)) keyword combinations from a primary keyword and a secondary keyword were searched in all titles, abstracts, keywords, and KeyWords Plus® fields. We limited our analysis to research articles or other forms of communication that presented new knowledge and were published between 1981 and 2018.

**Table 1:** Primary and secondary keywords used in publication searching on the Web of Science

Primary keywords	Concept	Secondary keywords
Gun, handgun, firearm, rifle, shotgun, pistol, revolver, semi-automatic, bullet, ammunition	Violence	violence, murder, homicide, assault, crime, lethal, defense, shooting, fatality, kill, massacre
	Perpetrator	criminal, felon, offender, shooter, perpetrator, crime
	Health outcome	death, suicide, mortality, gunshot, dead, die, wound, shot, fatal injury, fatality
	Risk factor	risk, safe, ownership store, storage, possess, conceal, lock, vault, control, cabinet, disable
	Law	Legislation, law, statute, regulation, concealed carry, background check, possession, domestic violence, surrender, removal, remove, sale, dealer, license, permit, waiting period, criminal history, revoke, revocation, renew, expire, policy, enforce, legislative, implement, liberty, loophole, punish, preliminary, register, access, administer, administration, Brady law, Brady bill, Heller, McDonald, 2nd Amendment, Second Amendment

### Subject category selection and review process

We examined trends in research related to gun violence by relying on Web of Sciences’ 254 subject categories (e.g., anthropology, biomedical engineering, ecology, and emergency medicine). Two researchers of this study independently determined whether journal articles within each subject category could potentially contain gun violence-related research. They then independently assigned “screened in” subject categories into the following disciplines: clinical sciences, life sciences, and social behavior sciences. In the event of discordance, a third reviewer broke the tie. We adopted the highest principle to assign a subject category in a group by reaching an agreement from the two reviewers. Thus, a total of 68 subject categories (26.77%) were assigned, where clinical sciences have 15, life sciences have 14, and social behavior sciences have 39 subject matter categories. The corresponding subject categories in each research discipline are contained in Table S1 (Additional file 1).

### Data processing

The original keywords query in the Web of Sciences returned 12,675 publications, including 3815 in clinical sciences, 3816 in life sciences, and 5044 in social behavior sciences. Because we found many duplicate publications in the original query, we de-duplicated to filter out replicated publications in each research discipline. We then excluded articles without keywords in the titles. These steps resulted in 1037 publications in clinical sciences, 914 publications in life sciences, and 1373 publications in social behavior sciences. Then, two researchers of this study independently screened article titles to determine if the publication was about gun violence and resolved any discordances through discussion. If the two researchers determined that an article was not gun violence research, a third researcher reviewed the article’s contents to verify that the article was indeed unrelated to gun violence research. After screening, 513 publications remained in clinical sciences, 544 publications remained in life sciences, and 843 publications remained in social behavioral sciences. We further excluded publications for which the study areas were outside the US after reviewing the contents of the articles. This exclusion resulted in 448 publications in clinical sciences, 537 in life sciences, and 812 in social behavior sciences.

### Statistical analysis

This study adopted two statistical modeling approaches. First, we applied the joinpoint regression approach (Kim et al. 2000) to test whether an apparent change in publication trend was statistically significant from 1981 to 2018 in each research discipline and the disciplines combined. The approach can evaluate the number of joinpoints to partition the trend into several segments, and the change in each segment can be estimated by the following model equation:

$$ \mathsf{Log}\left({\mathsf{NP}}_{\mathsf{t}}\right)=\mathsf{\alpha}+\mathsf{\beta}\times \mathsf{t}+\mathsf{\log}\left({\mathsf{Total}}_{\mathsf{t}}\right),\mathsf{where}\ \mathsf{t}=\mathsf{1},\dots, \mathsf{38} $$

, where NP_t_ is the number of publications in gun violence research at calendar time t, and log(Total_t_) is the offset from the annual total publications. A model selection was adopted to choose a joinpoint regression from 0 to 4 joinpoints, and the best model was determined by the smallest Bayesian information criterion (BIC). Because the whole study period is across four decades, we limited the maximum number of joinpoints by four to prevent short segments. The annual percent change (APC) of each segment and the average annual percent change (AAPC) of the entire study period were also calculated based on the best model.

Second, we built a generalized additive mixed model to compare the differences of publication ratios among the three research disciplines, and evaluated the influence of gun ownership and firearm homicide and suicide death on publication ratios. Suppose the number of publication of research discipline i (1 = clinical sciences; 2 = life sciences; 3 = social behavior sciences) at time t is denoted by NP_it_, which follows a Poisson distribution POI(μ_it_), where μ_it_ is the mean of NP_it_, the model equation can be built as:

$$ \mathsf{Log}\left({\mathsf{NP}}_{\mathsf{i}\mathsf{t}}\right)=\mathsf{\alpha}+{\mathsf{\alpha}}_{\mathsf{i}\left(\mathsf{t}\right)}+{\mathsf{\beta}}_{\mathsf{1}}\times \mathsf{I}\left(\mathsf{i}=\mathsf{1}\right)+{\mathsf{\beta}}_{\mathsf{2}}\times \mathsf{I}\left(\mathsf{i}=\mathsf{2}\right)+\mathsf{f}\left(\mathsf{t}\right)+\mathsf{\log}\left({\mathsf{Total}}_{\mathsf{i}\mathsf{t}}\right),\mathsf{where}\ \mathsf{i}=\mathsf{1},\mathsf{2},\mathsf{3};\mathsf{t}=\mathsf{1},\dots, \mathsf{3}\mathsf{8} $$

, where α is a fixed intercept, and α_i_ is a random intercept specified by research disciplines nested with time, which can account for unobserved predictors affecting the number of publications. The symbols β_1_ and β_2_ are the coefficient parameters of two indicator functions for clinical sciences (i.e., I(i = 1)) and life sciences (i.e., I(i = 2)), respectively, as social behavior research is defined as the reference level. The autocorrelation is controlled by a time smoothing spline f(t), where its smoothing parameter is estimated by generalized cross validation. The estimated β_1_ and β_2_ were transformed into rate ratios to compare the publication ratio of clinical sciences and life sciences versus social behavior sciences. The last term log(Total_it_) is an offset calculated from the logarithm of the total number of publications in each research discipline at time t.

Statistical analyses were implemented by Joinpoint Regression Program V.4.7.0.0 (National Cancer Institute, USA) and SAS v9.4 (SAS Institute Inc., Cary). The significance level was set to 5%.

## Results

**Fig. 1:**
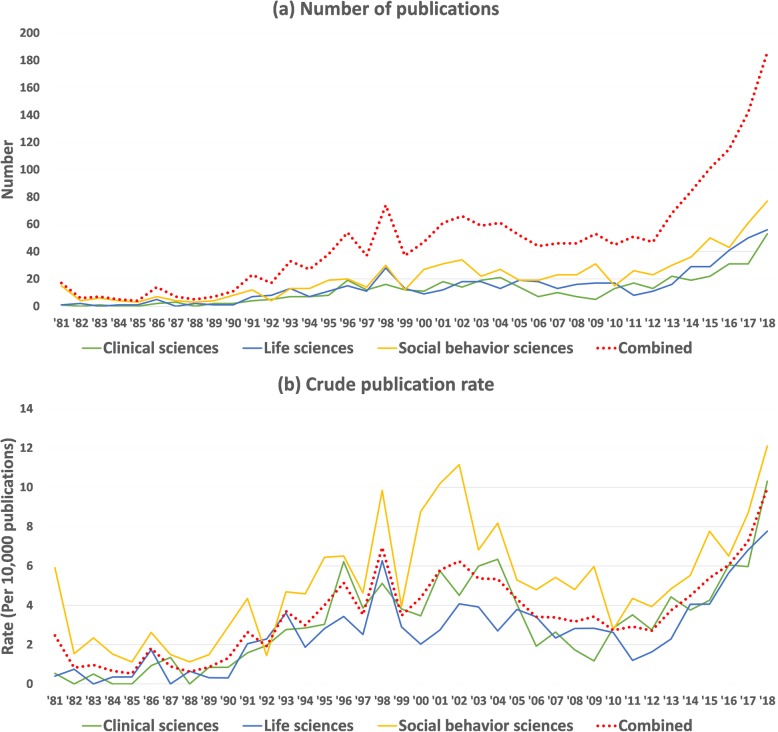
Time trends of the number of publications and crude publication ratios by research discipline from 1981 to 2018

Figure 1 shows certain variations in both the number of publications and the crude publication ratio of gun violence research in each discipline. Social behavioral sciences had more related publications and a higher publication ratio in most study years. As Table 2 indicates, in the social behavioral sciences discipline, on average, 21.37 papers about gun violence were published per year (Standard deviation = 16.46), which is more than the annual average of clinical sciences and life sciences. Moreover, social behavioral sciences had the highest publication ratio (51.68 per 100,000 publications) compared to the other two research disciplines. Furthermore, even though life sciences had more publications than clinical sciences per year (14.13 vs. 11.79), its annual publication ratio was smaller (26.14 vs. 30.97 per 100,000 publications).

**Table 2:** Summary statistics of gun violence publications by research discipline, 1981–2018

Research discipline	Mean	STD	Min	Q1	Q3	Max
Number of total publications						
Clinical sciences	33,251.84	10,962.44	18,523.00	23,957.75	42,096.75	51,939.00
Life sciences	46,820.47	15,772.21	25,186.00	32,982.00	59,231.75	73,430.00
Social behavior sciences	38,584.76	14,907.55	25,367.00	27,526.75	50,894.75	70,257.00
Combined	118,657.08	41,250.54	69,076.00	84,534.50	152,223.25	195,626.00
Number of gun violence publications						
Clinical sciences	11.79	10.90	0.00	3.25	17.75	53.00
Life sciences	14.13	13.12	0.00	5.50	17.75	56.00
Social behavior sciences	21.37	16.46	3.00	9.00	29.25	77.00
Combined	42.55	35.39	4.00	16.00	53.00	160.00
Crude publication ratio (per 100,000 publications)						
Clinical sciences	30.97	23.10	0.00	12.15	43.86	103.12
Life sciences	26.14	18.79	0.00	13.08	35.64	77.71
Social behavior sciences	51.68	29.07	11.17	28.04	65.10	121.02
Combined	32.03	19.24	4.99	18.15	46.68	85.53

Table 3 shows the BIC among different joinpoints in terms of individual research disciplines and the combination of disciplines. In individual research disciplines, both clinical sciences and social behavioral sciences had the best model with three joinpoints, where their smallest BIC was 0.40 and 1.05, respectively. The best model for life sciences only had two joinpoints with the smallest BIC = 1.01. Considering the combination of the three research disciplines, the model with two joinpoints was determined the best model with the smallest BIC = 1.45.

**Table 3:** The Bayesian information criterion (BIC) in the joinpoint regression. The asterisk represents the best model with the smallest BIC in each research discipline

Model	# of Joinpoints	Clinical sciences	Life sciences	Social behavior sciences	Combined
#1	0 Joinpoint	1.60	1.84	2.26	2.54
#2	1 Joinpoint	1.45	1.71	2.21	2.52
#3	2 Joinpoints	0.53	0.86*	1.32	1.43
#4	3 Joinpoints	0.17*	0.98	0.79*	1.23
#5	4 Joinpoints	0.24	1.08	0.91	1.19*

**Fig. 2:**
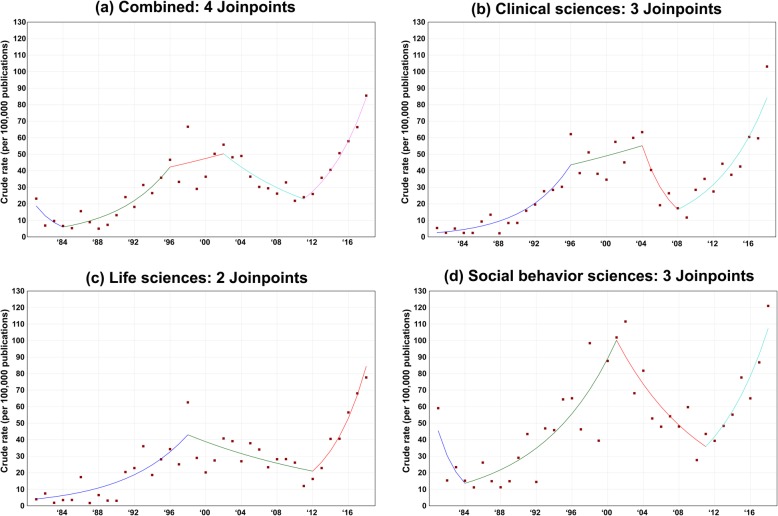
Time trends of publication ratios with joinpoints and the annual percentage change in each segment from 1981 to 2018. The asterisks shown in the legends indicate that the annual percentage change is significantly different from 0 with a *p*-value < 0.05

The selected joinpoints, shown in Fig. 2, explicitly reveal an increasing trend before 1998 in each research discipline, except for social behavior sciences from 1981 to 1984. Moreover, after 1998 each specific research discipline had at least one decreased APC in the publication ratio. Table 4 shows that those decreased trends significantly appeared from 1998 to 2012 in life sciences (APC = − 4.99%; 95% confidence interval (CI) = − 8.23, − 1.64; *p*-value = 0.0052) and from 2001 to 2011 in social behavior sciences (APC = − 9.77%; 95% CI = − 13.45, − 5.93; *p*-value < .0001). In clinical sciences, the decreased trend was shorter (2004–2008) and not significant. The decreased trend was first bounced back in clinical sciences since 2008 (APC = 17.65%; 95% CI = 13.96, 21.46; *p*-value < .0001), and then in social behavior sciences since 2011 (APC = 16.96%; 95% CI = 11.35, 22.85; *p*-value < .0001). Even though the APC in life sciences did not increase until 2012, it was 26.10% (95% CI = 16.77, 36.17; *p*-value < .0001), the largest significant increase, compared to the other two research disciplines. Combining the three research disciplines,. the total publication ratio had two significant increases from 1984 to 1996 (APC = 17.76%; 95% CI = 11.14, 24.76; *p*-value < .0001) and from 2011 to 2018 (APC = 20.44%; 95% CI = 15.06, 26.07; *p*-value < .0001). However, a significant decrease was observed from 2002 to 2011 (APC = − 8.37%; 95% CI = − 12.84, − 3.68; *p*-value < .0001).

**Table 4:** The annual percentage change (APC) of the publication ratio in gun violence studies from 1981 to 2018

Lower year	Upper year	APC	95% confidence interval	***P***-value
		**Clinical sciences**		
1981	1996	20.86	(14.62, 27.43)	<.0001
1996	2004	2.99	(-3.36, 9.76)	0.3500
2004	2008	-25.99	(-45.58, 0.65)	0.0546
2008	2018	17.65	(13.96, 21.46)	<.0001
		**Life sciences**		
1981	1998	14.76	(9.50, 20.28)	<.0001
1998	2012	-4.99	(-8.23, -1.64)	0.0052
2012	2018	26.10	(16.77, 36.17)	<.0001
		**Social behavior sciences**		
1981	1984	-33.04	(-58.75, 8.70)	0.1009
1984	2001	12.44	(9.33, 15.64)	<.0001
2001	2011	-9.77	(-13.45, -5.93)	<.0001
2011	2018	16.96	(11.35, 22.85)	<.0001
		**Combined**		
1981	1984	-31.77	(-61.66, 21.42)	0.1837
1984	1996	17.76	(11.14, 24.76)	<.0001
1996	2002	2.95	(-7.55, 14.63)	0.5825
2002	2011	-8.37	(-12.84, -3.68)	0.0014
2011	2018	20.44	(15.06, 26.07)	<.0001

From 1981 to 2018, the average change of the publication ratio in the combined research disciplines per year was significantly increased with an AAPC = 9.18% (95% CI = 6.42, 12.01; p-value < .0001). In the individual research disciplines, the AAPC was the highest in clinical sciences, where the publication ratio significantly increased 9.92% per year (95% CI = 5.52, 14.50; *p*-value < .0001). Life sciences had a lower but still significant AAPC = 8.49% (95% CI = 5.60, 11.46; *p*-value < .0001). No significant AAPC was found in social behavioral sciences.

The publication ratio had significant differences across research disciplines after controlling for gun ownership, firearm homicide, and firearm suicide rates. Compared to social behavioral sciences, clinical sciences had a significantly smaller publication ratio by 0.64 times (95% CI = 0.55, 0.73; p-value < .0001). In addition, the publication ratio in life sciences was 0.54 times (95% CI = 0.47, 0.61; p-value < .0001), significantly fewer than that in social behavioral sciences.

## Discussion

This study bolsters the existing literature about gun violence research. We used bibliometric analysis to analyze variation in gun violence-related research in three discipline areas between 1981 and 2018. We found that all disciplines followed a similar pattern, with two increases bisected by a decrease. The late 1990’s/early 2000’s began a decade long decrease in gun violence related research publications. Previous studies have tracked the prevalence of gun violence research over time (Stark and Shah 2017; Ladapo et al. 2013; Alcorn 2017) and have demonstrated that gun violence research has been relatively limited both in quantity and relative the impact of gun violence on health (Stark and Shah 2017; Ladapo et al. 2013). This study adds to the literature by identifying points of significant increase and decrease in gun violence research over time across disciplines. It reaches these findings through systematic searching and screening of published literature in multiple databases across disciplines over four decades and applying a statistical model with sound methodology to evaluate changes over time.

The findings of this study are important for understanding past and contemporary policy choices and their effects. This study lends analytical support for links made in the literature suggesting a direct relationship between the availability of federal funding for gun violence research and peer-reviewed publications (Stark and Shah 2017; Ladapo et al. 2013; Alcorn 2017). This study found meaningful increases in research in 1984 and 2011 and a meaningful decrease in 2002. The observed decrease is consistent with certain federal funding patterns for health-related gun violence research. The Dickey Amendment, which if not in substance at least in effect halted CDC-funded research in this area, was originally adopted in 1996 and subsequently re-authorized. Similar limitations also applied to the National Institute of Health (NIH) in 2011 (Rostron 2018). The decrease we observed in overall publications in 2002 may be explained by the cumulative effect of the Dickey Amendment given, at least in part, the typical lag between when federal research funding is awarded and when resulting peer-reviewed research is ultimately published. In our study, the significant decrease in the disciplines of life sciences and social and behavioral health are especially consistent with this explanation since research in these disciplines can be funded by the US Department of Health and Human Services (HHS). Interestingly, clinical sciences, also often funded by HHS, saw less of a decrease at this time. This may be explained in part by the adoption of alternate funding stipulations on the NIH, which funds much more clinical research than the CDC.

This study is not without limitations. First, we used only one database, Web of Science, which may have missed relevant articles. We also only analyzed research in three discipline areas, though these three areas did have the highest amount of publications related to firearm violence. While a strength of our study is that we screened each article title to ensure it was firearm-related, it is possible that we may have missed some relevant articles that did not make apparent their connection to firearm violence through their titles. Therefore, it is possible that relevant articles were missed. We also only considered articles that were examining topics specific to the US. While the US has the highest firearm mortality rates in the world, and very unique policy and funding related issues, a more global picture of firearm research may be beneficial. Furthermore, two researchers assigned subject categories to research. It is possible that outside researchers would assign some subject categories to different research disciplines. To minimize this bias, however, the researchers assigned subject categories to research disciplines independently and in the event of discordance, a third reviewer broke the tie. In addition, if an article was categorized into multiple subject categories by Web of Science and these subject categories were assigned to different research disciplines, the article would appear multiple times in our results. Counting them twice means some duplicate articles across research categories; but it also means that each research category received “credit” for such an article.

Our findings are consistent with the notion that limited funding tracks with gun violence publication across research disciplines. Gun violence has remained a problem in the U.S. during the time period of study but publication rates seemed to shift. Our findings of specific upward and downward trends in publication sets the stage for future inferential work that examine the exact determinants of these changes. Monitoring both funding and publication of gun violence research is especially important if federal funding truly becomes more readily available for this type of work. Such monitoring could include systematically gathering all kinds of research resources (e.g., data, funding opportunities, publications) in an open platform to facilitate accessing necessary information to develop gun violence research. A recent good news is that federal agencies will receive $25 million from Congress to study gun violence, including $12.5 million each for the CDC and NIH (Hellmann 2019). This is the first time after 1996 when Congress stopped funding gun violence research according to the Dickey Amendment. We hope the upward trend in the past 10 years revealed by this study along with the future’s federal funding sources will motivate more researchers, no matter experienced or new blood, to bring an optimistic prospect in gun violence research area.

## Conclusions

Research and the search for knowledge may be intrinsically worthwhile. In public health, however, research is also critical to understand the scope of a problem, its risk factors, and the effectiveness of potential interventions - including programs, policies, and laws. Approaching gun violence as a public health problem therefore requires producing quality research that identifies the scope of the problem and potential solutions and then utilizing this research in practice. Without a robust body of gun violence research, the effectiveness of gun violence interventions is a function of trial and error. This study supports the notion that the aggregate amount of gun violence research is sensitive to the context in which it is completed. Such context might be research funding and gun violence trends. Thus, to enhance the amount of peer-reviewed gun violence research so that research-informed gun violence interventions are more likely to succeed, decision-makers should keep supporting quality research.

## Supplementary information

**Additional file 1.** Table S1. Selected subject categories by research discipline.

## Data Availability

The datasets generated during and analyzed during the current study are available from the corresponding author on reasonable request.

## Abbreviations

AAPC: Average annual percent change
APC: Annual percent change
BIC: Bayesian information criterion
CDC: Centers for disease control and prevention
