# Correction to: Temporal trend of research related to gun violence from 1981 to 2018 in the United States: a bibliometric analysis

**DOI:** 10.1186/s40621-020-00266-x

**Published:** 2020-07-13

**Authors:** Lung-Chang Chien, Maxim Gakh, Courtney Coughenour, Ro-Ting Lin

**Affiliations:** 1 Department of Environmental and Occupational Health, School of Public Health, University of Nevada, Las Vegas, 4700 S. Maryland Pkwy, Suite 335, Las Vegas, Nevada 89119 USA; 2 Department of Occupational Safety and Health, College of Public Health, China Medical University, No. 91, Xueshi Road, North District, Taichung City, 404 Taiwan

**Correction to: Inj Epidemiol (2020) 7:9**

**https://doi.org/10.1186/s40621-020-0235-6**

Following publication of the original article (Chien et al. 2020), the authors identified two errors;

1. The equations were not adequately presented since they all Greek symbols disappeared in the PDF version of the article.
  The correct presentation of the equations is given below.

$$ \mathrm{Log}\left({\mathrm{NP}}_{\mathrm{t}}\right)=\alpha +\beta \times \kern0.5em \mathrm{t}+\log \left({\mathrm{Total}}_{\mathrm{t}}\right),\mathrm{where}\kern0.5em \mathrm{t}=1,\dots, 38 $$

$$ \mathrm{Log}\left({\mathrm{NP}}_{\mathsf{i}\mathsf{t}}\right)=\upalpha +{\upalpha}_{\mathsf{i}\left(\mathsf{t}\right)}+{\upbeta}_{\mathsf{1}}\times \mathsf{I}\left(\mathsf{i}=\mathsf{1}\right)+{\upbeta}_2\times \mathrm{I}\left(\mathrm{i}=2\right)+\mathrm{f}\left(\mathrm{t}\right)+\log \left({\mathrm{Total}}_{\mathrm{it}}\right),\mathrm{where}\ \mathrm{i}=1,2,3;\mathrm{t}=1,\dots, 38 $$

2) The grey horizontal lines were missing in Fig. 1. The correct figure is given below.

**Fig. 1:**
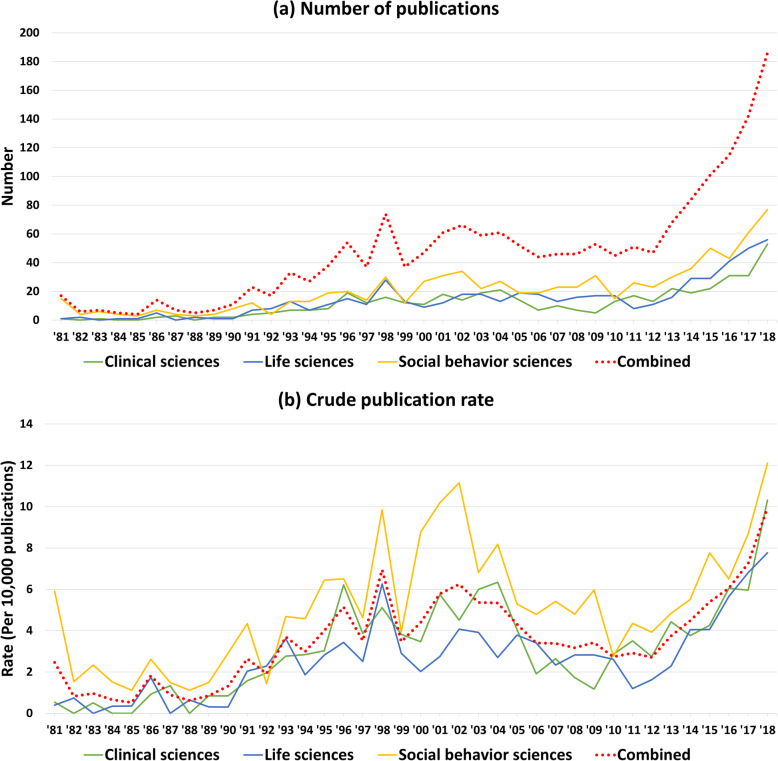
Time trends of the number of publications and crude publication ratios by research discipline from 1981 to 2018

The original article (Chien et al. 2020) has been updated.
