# Supplementary material for: Temporal trend of research related to gun violence from 1981 to 2018 in the United States: a bibliometric analysis
Source: Inj Epidemiol. 2020 Mar 23;7:9. doi: 10.1186/s40621-020-0235-6 (PMC7087386; doi:10.1186/s40621-020-0235-6)
Supplement: Supplementary file 1 — Additional file 1. Table S1. Selected subject categories by research discipline. [file 40621_2020_235_MOESM1_ESM.docx]

**Additional File**

Temporal trend of research related to gun violence from 1981 to 2018 in the United States: A bibliometric analysis

Lung-Chang Chien^1,*^, Maxim Gakh^1^, Courtney Coughenour^1^, Ro-Ting Lin^2^

^1^ Department of Environmental and Occupational Health, University of Nevada, Las Vegas, School of Public Health, Nevada, USA

^2^ Department of Occupational Safety and Health, China Medical University, College of Public Health, Taiwan

*Corresponding author:

Lung-Chang Chien, Dr.PH

Department of Environmental and Occupational Health

University of Nevada, Las Vegas

School of Public Health

Address: 4505 S. Maryland Pkwy, Box 453064, Las Vegas, Nevada 89154

Phone: +1-702-895-5282

Email: [lung-chang.chien@unlv.edu](mailto:lung-chang.chien@unlv.edu)

Table S1. Selected subject categories by research discipline

| **Clinical sciences** | **Life sciences** | **Social behavioral sciences** |
| --- | --- | --- |
| Anesthesiology  Cardiac & Cardiovascular Systems  Clinical Neurology  Critical Care Medicine  Dentistry, Oral Surgery & Medicine  Emergency Medicine  Gastroenterology & Hepatology  Geriatrics & Gerontology  Hematology  Integrative & Complementary Medicine  Medicine, General & Internal  Pediatrics  Primary Health Care  Psychiatry  Surgery | Biology  Biophysics  Biotechnology & Applied Microbiology  Environmental Studies  Genetics & Heredity  Medicine, Research & Experimental  Neurosciences  Nursing  Pathology  Pharmacology & Pharmacy  Physiology  Public, Environmental & Occupational Health  Sport Sciences  Toxicology | Anthropology  Behavioral Sciences  Communication  Criminology & Penology  Demography  Economics  Education & Educational Research  Education, Scientific Disciplines  Education, Special  Family Studies  Geography  Geography, Physical  Gerontology  Law  Medicine, Legal  Multidisciplinary Sciences  Political Science  Psychology  Psychology, Applied  Psychology, Biological  Psychology, Clinical  Psychology, Developmental  Psychology, Educational  Psychology, Experimental  Psychology, Mathematical  Psychology, Multidisciplinary  Psychology, Psychoanalysis  Psychology, Social  Public Administration  Regional & Urban Planning  Social Issues  Social Sciences, Biomedical  Social Sciences, Interdisciplinary  Social Sciences, Mathematical Methods  Social Work  Sociology  Substance Abuse  Telecommunications  Urban Studies |
